# Anxiety, Depression, And PTSD Among A Cohort Of Middle Aged And Older Transgender Adults

**DOI:** 10.1093/geroni/igaf122.2398

**Published:** 2025-12-31

**Authors:** Ethan Cicero, Jace Flatt, Vin Tangpricha, Darios Getahun, Courtney McCracken, Michael Silverberg, Suma Vupputuri, Michael Goodman

**Affiliations:** Emory University, Atlanta, Georgia, United States; University of Nevada Las Vegas, Nevada, United States; Emory University, Atlanta, Georgia, United States; Kaiser Permanente Southern California, Pasadena, California, United States; Kaiser Permanente Georgia, Atlanta, Georgia, United States; Kaiser Permanente Northern California, Oakland, California, United States; Kaiser Permanente Mid-Atlantic States, Washington, District of Columbia, United States; Emory University, Atlanta, Georgia, United States

## Abstract

Systematic studies of mental health status among older transgender adults are largely lacking. Kaiser electronic health records (01/2006–03/2023) were used to identify a cohort of 2,362 transfeminine (TF) and 1,185 transmasculine (TM) individuals aged 45+ years enrolled in three integrated health systems. Each transgender individual was matched to 10 cisgender men (CM) and 10 cisgender women (CW) on birth year, race/ethnicity, study site, and enrollment at index date (first evidence of transgender status in TF/TM cohort members). Enrollment time-adjusted odds ratios (aOR) along with 95% confidence intervals (CI) were calculated to examine prevalence of anxiety, depression, and post-traumatic stress disorder (PTSD) among TF and TM adults compared to their respective CM and CW referents. In the analyses comparing TF cohort members to CW referents the aOR (95% CI) estimates were 2.01 (1.84–2.20) for anxiety, 2.66 (2.43–2.90) for depression, and 3.27 (2.80–3.81) for PTSD. When TF individuals were compared to CM referents the aOR was 4.13 (3.77–4.52) for anxiety, 5.46 (4.99–5.97) for depression, and 7.53 (6.33–8.96) for PTSD. The aORs were more pronounced for TM relative to CM and attenuated for TM relative to CW. All associations were stronger in the sub-analyses restricted to persons of color whereas the sub-analyses focusing on gender-affirming hormone therapy recipients showed no discernable difference from the overall results. Older TF and TM adults experience a higher burden of mental health conditions compared to their cisgender counterparts. This burden appears to disproportionally affect transgender persons of color.

